# Use of Social Media in the Diabetes Community: An Exploratory Analysis of Diabetes-Related Tweets

**DOI:** 10.2196/diabetes.6256

**Published:** 2016-11-07

**Authors:** Yang Liu, Qiaozhu Mei, David A Hanauer, Kai Zheng, Joyce M Lee

**Affiliations:** 1 School of Information University of Michigan Ann Arbor, MI United States; 2 Department of Electrical Engineering and Computer Science College of Engineering University of Michigan Ann Arbor, MI United States; 3 Department of Pediatrics Medical School University of Michigan Ann Arbor, MI United States; 4 Department of Health Management and Policy School of Public Health University of Michigan Ann Arbor, MI United States; 5 Department of Pediatric Endocrinology Medical School University of Michigan Ann Arbor, MI United States; 6 Child Health Evaluation and Research Center University of Michigan Ann Arbor, MI United States

**Keywords:** social media, Twitter, DSMA, diabetes community, spatiotemporal analysis, content analysis

## Abstract

**Background:**

Use of social media is becoming ubiquitous, and disease-related communities are forming online, including communities of interest around diabetes.

**Objective:**

Our objective was to examine diabetes-related participation on Twitter by describing the frequency and timing of diabetes-related tweets, the geography of tweets, and the types of participants over a 2-year sample of 10% of all tweets.

**Methods:**

We identified tweets with diabetes-related search terms and hashtags in a dataset of 29.6 billion tweets for the years 2013 and 2014 and extracted the text, time, location, retweet, and user information. We assessed the frequencies of tweets used across different search terms and hashtags by month and day of week and, for tweets that provided location information, by country. We also performed these analyses for a subset of tweets that used the hashtag #dsma, a social media advocacy community focused on diabetes. Random samples of user profiles in the 2 groups were also drawn and reviewed to understand the types of stakeholders participating online.

**Results:**

We found 1,368,575 diabetes-related tweets based on diabetes-related terms and hashtags. There was a seasonality to tweets; a higher proportion occurred during the month of November, which is when World Diabetes Day occurs. The subset of tweets with the #dsma were most frequent on Thursdays (coordinated universal time), which is consistent with the timing of a weekly chat organized by this online community. Approximately 2% of tweets carried geolocation information and were most prominent in the United States (on the east and west coasts), followed by Indonesia and the United Kingdom. For the user profiles randomly selected among overall tweets, we could not identify a relationship to diabetes for the majority of users; for the profiles using the #dsma hashtag, we found that patients with type 1 diabetes and their caregivers represented the largest proportion of individuals.

**Conclusions:**

Twitter is increasingly becoming a space for online conversations about diabetes. Further qualitative and quantitative content analysis is needed to understand the nature and purpose of these conversations.

## Introduction

Use of social media is becoming ubiquitous among US individuals; according to the Pew Research Center, at least 76% of adults who are Internet users use some form of social networking site such as Facebook or Twitter [1]. Surveys have shown that 7 out of 10 US adults with chronic diseases are (1) looking online for health information about medical problems, treatments, and drugs; (2) consulting online reviews about treatments; and (3) learning about others’ personal health experiences [2].

Although Facebook is still the most popular social media channel, use of additional social media channels in the US population is increasing; for example, in 2014 an estimated 23% of online adults reported that they use Twitter [3]. In particular, patients and caregivers with diabetes started to congregate and participate in online conversations about diabetes on Twitter [4], engage in virtual communication and sharing, and find peer support online.

There is growing interest in studying disease-related communities of interest online. Studies in the scientific literature have analyzed content of a small number of tweets within a short time period; for example, studies have looked at the use of Twitter by local health departments for dissemination of information about diabetes [5,6] and have performed content analysis and user profile classification with hundreds of diabetes conversations on Twitter [7,8], but we are unaware of research studies that have formally tried to perform large-scale evaluation of Twitter metrics among communities of interest focused on diabetes.

Our objective was to examine diabetes-related participation on Twitter by describing the frequency and timing of diabetes-related tweets, the geography of tweets, and the types of participants over a 2-year sample of 10% of all tweets. The results will help us better understand the extent to which patients, caregivers, and medical practitioners participate in social media discussions related to diabetes.

## Methods

### Data Collection

We used a dataset that contains 29.6 billion tweets obtained during 2013 and 2014 collected through the Twitter stream application programming interface (API) with Gardenhose access, which collects 10% of all public communications on Twitter (secured through a formal agreement with the University of Michigan School of Information). We identified tweets with diabetes-related search terms and hashtags based on suggestions from providers and patients in the diabetes community using the following query terms and hashtags: “glucose,” “blood glucose,” “diabetes,” “insulin pump,” “insulin,” “#diabetes,” “#t1d,” “#type1diabetes,” “#type1,” “#t2d,” “#type2diabetes,” “#type2,” “#bloodsugar,” “#dsma” (Diabetes Social Media Advocacy is an online advocacy group which holds a weekly “tweetchat” to provide peer support to individuals with diabetes), “#doc” (diabetes online community), “#bgnow” (blood glucose now, in which individuals share their blood sugars), “#wearenotwaiting” (a phrase coined by the diabetes community related to the need for rapid access to technology solutions), “#showmeyourpump” (a tweet campaign that occurred when a Miss America contestant decided to wear her insulin pump visibly), “CWD2014” (children with diabetes, a diabetes conference for children and families with diabetes), “dblog”(diabetes blog), and “diyps” (a do-it-yourself artificial pancreas project).

### Spatiotemporal Analysis

For each tweet retrieved, we extracted its text content, the username of the tweet, the tweet’s posted date and time, the geolocation information of the tweet if available, and whether the tweet is a retweet. We assessed the frequencies with which the retrieved tweets are used across different terms and hashtags. With the posted date information of each tweet, we examined the trend of volume of extracted tweets in each month. We conducted an analysis with 2 subsets: all users with a diabetes-related tweet and users who posted at least once with the #dsma hashtag.

### User Identities Analysis

We then examined the identities of two subsets of users. We randomly sampled 500 users from the entire dataset. There were 1424 individuals who had tweeted at least once with the hashtag #dsma; we chose to focus on a smaller subset, those who had tweeted at least 3 times with hashtag #dsma (n=416), because it would identify more active members of the community and it represented a sample similar in number to our overall diabetes sample. A medical student reviewed each of the Twitter profiles to identify individuals’ relationship to diabetes, which was categorized into one or more of the following 15 categories: physician, nurse, dietitian, diabetes educator, researcher, individual with type 1 diabetes, individual with type 2 diabetes, individual with diabetes not specified, caregiver/parent/guardian of an individual with diabetes, spouse/significant other of an individual with diabetes, friend of an individual with diabetes, individual who works with a diabetes-related company, health care organization, diabetes medical/device company, and other/unknown. A second individual reviewed another 50 randomly selected profiles for both subsets of users. There was interrater agreement on 44 of the 50 categorizations for the all-user subset. The Cohen kappa was .58. In the subset of #dsma users, there was interrater agreement on 40 of the 50. The Cohen kappa was .71.

## Results

Of the 29.6 billion tweets in our entire dataset, there were 1,368,575 diabetes-related tweets, based on the selected diabetes terms and hashtags. One-third of these tweets (454,261) were retweets.

Table 1 shows the number and percentage of tweets and the number and percentage of users tweeting with specific search terms or hashtags in our dataset. The most common tweets were the terms including the term or hashtag diabetes, followed by insulin and glucose, and then finally references to the type of diabetes (ie, type 1 or type 2 diabetes).

**Table 1:** Number and percentage of tweets by terms or hashtags and the number and percentage of users tweeting with those terms or hashtags.

Term	Tweets n (%)	Users n (%)
Diabetes	1,200,268 (87.7)	748,001 (89.6)
#Diabetes	165,868 (12.1)	67,229 (8.1)
Insulin	83,820 (6.1)	59,728 (7.2)
Glucose	60,033 (4.4)	46,357 (5.6)
#Doc	27,616 (2.0)	16,457 (2.0)
#Dsma	11,757 (0.9)	1424 (0.2)
Blood glucose	10,212 (0.7)	6904 (0.8)
#T1d	9040 (0.7)	3835 (0.5)
#Dblog	5711 (0.4)	1132 (0.1)
Insulin pump	5179 (0.4)	4061 (0.5)
#Type1	3211 (0.2)	1800 (0.2)
#Type2	2905 (0.2)	1468 (0.2)
#Bgnow	2470 (0.2)	753 (0.1)
#Type1diabetes	1812 (0.1)	1248 (0.1)
#Bloodsugar	1718 (0.1)	1213 (0.1)
#Type2diabetes	1388 (0.1)	1035 (0.1)
#T2d	935 (0.1)	452 (0.1)
#Showmeyourpump	932 (0.1)	645 (0.1)
#Wearenotwaiting	327 (<0.1)	183 (<0.1)
#Diyps	132 (<0.1)	50 (<0.1)
#Cwd2014	7 (<0.1)	7 (<0.1)

**Figure 1:**
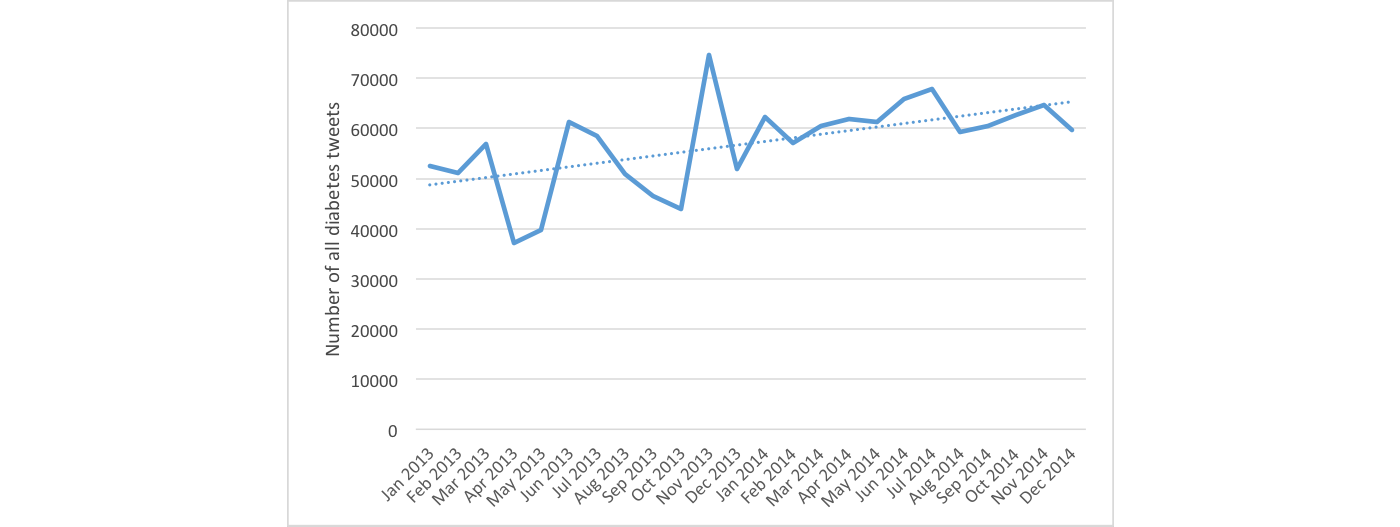
Timeline of tweet volume for all diabetes-related tweets.

**Figure 2:**
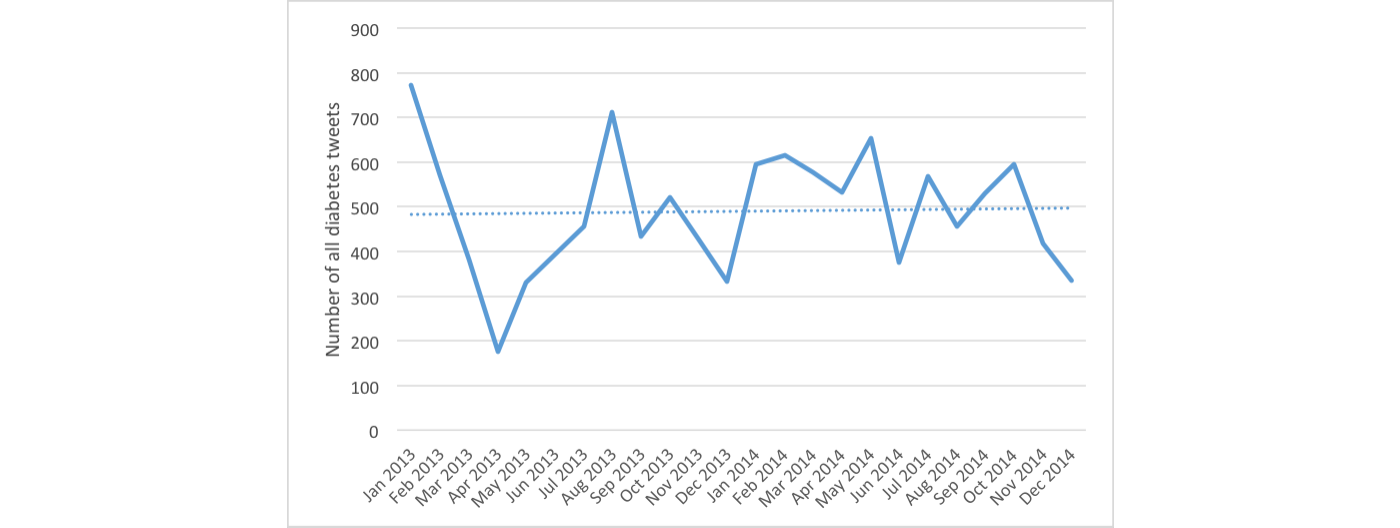
Timeline of tweet volume for tweets using the hashtag #dsma.

Figure 1 shows the monthly breakdown of diabetes-related tweets over the 2-year period. The peak occurred in November 2013 on World Diabetes Day, with over 70,000 diabetes-related tweets (representing 10% of tweets). Figure 2 shows the total number of tweets for community using the #dsma hashtag. Both figures show increasing trends of the tweets volume.

**Figure 3:**
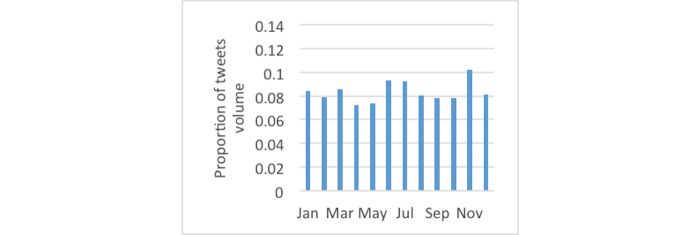
The proportion of tweets by month across the 2-year period for all diabetes-related tweets.

**Figure 4:**
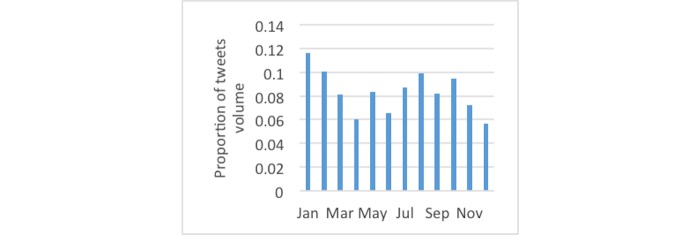
The proportion of tweets by month across the 2-year period for #dsma tweets.

**Figure 5:**
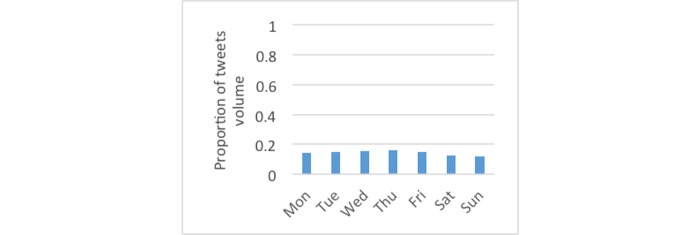
The proportion of tweets by day of the week across the 2-year period for all diabetes-related tweets.

**Figure 6:**
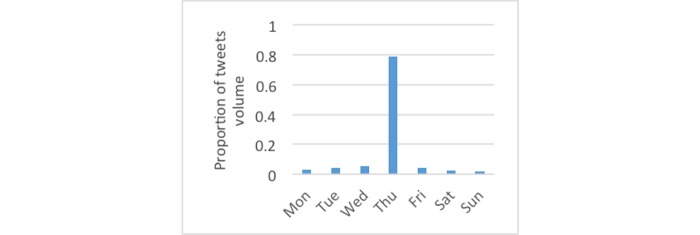
The proportion of tweets by day of the week across the 2-year period for #dsma tweets.

Figure 3 shows the monthly distributions of diabetes-related tweets, which were most frequent in November, likely attributable to World Diabetes Day. For tweets using the #dsma hashtag, Figure 4 shows January had the largest proportion. Figures 5 and 6 show that the proportion of diabetes-related tweets was higher during the weekdays compared with the weekend days; mean tweets per weekday were significantly higher than for weekends (2011 per weekday vs 1684 per weekend, *P*<.001). In contrast, the majority of #dsma tweets were posted on Thursdays (Twitter API returns coordinated universal time) due to the fact that there is an online chat organized by a community of individuals with diabetes and caregivers that uses the #dsma hashtag for participating in the conversations at 9 PM eastern standard time every Wednesday night.

**Figure 7:**
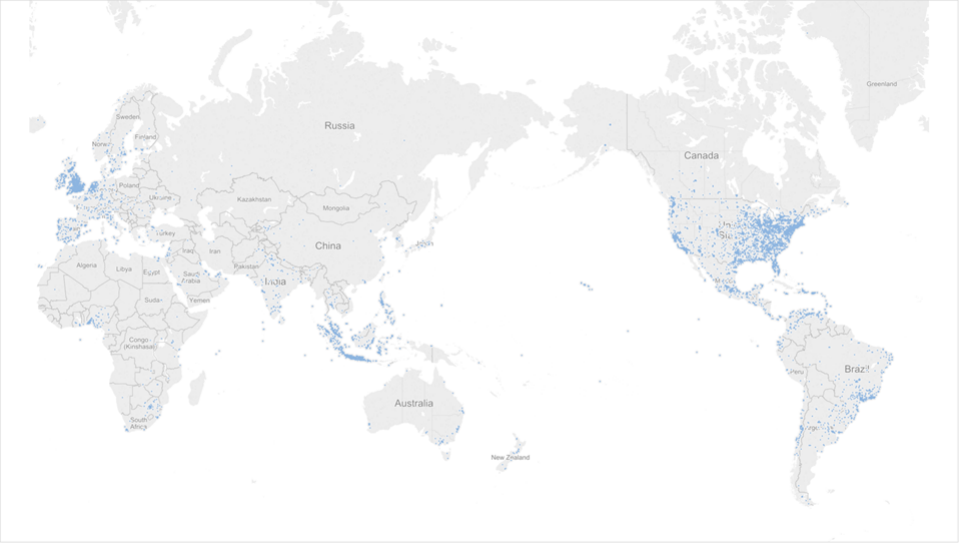
Visualization of diabetes-related tweets with geolocation information. Each blue dot represents one tweet.

Approximately 2% (26,763) of tweets carried geolocation information. Table 2 shows the number of geotagged tweets for countries with at least 100 geotagged tweets, which would likely bias toward English-speaking countries because of our query terms. The United States ranked first, followed by Indonesia, United Kingdom, Venezuela, and Mexico. Figure 7 displays the locations of the geotagged tweets on a world map. For the United States, the participation appeared to be located particularly on the east coast and midwest with pockets on the west coast.

Of the 500 users randomly selected from the diabetes-related tweets, 471 of them were categorized as other/unknown. Table 3 shows the breakdown of categories. There were just 29 users for whom an identity could be assigned, including 12 health care organizations and a handful of patients with type 1 or type 2 diabetes and health care providers. In contrast, only 15.6% of #dsma members’ identities were either not related to diabetes or unknown based on their Twitter profile information. The majority of individuals tweeting with #dsma had type 1 or type 2 diabetes or were caregivers. A very small percentage of individuals were health care professionals, and there was less company and health care stakeholder participation than with the general diabetes-related tweets.

**Table 2:** Frequency of the geotagged diabetes tweets in countries with more than 100 appearances.

Country	Number of geotagged diabetes tweets
United States	10,047
Indonesia	5355
United Kingdom	1897
Venezuela	1172
Mexico	1042
Brazil	816
Malaysia	611
Canada	590
Philippines	439
Ghana	350
Spain	325
Nigeria	299
Argentina	260
Chile	223
India	220
Australia	218
Dominican Republic	199
Netherlands	189
South Africa	185
Colombia	167
Singapore	147
Ireland	107
Sweden	105

**Table 3:** Categories of individuals who tweeted with diabetes-related tweets and #dsma tweets.

Users’ relationship to diabetes	Users who have posted diabetes-related tweets n (%)	Users who have posted #dsma tweets n (%)
Individual with type 1 diabetes	2 (0.4)	220 (52.9)
Individual with type 2 diabetes	1 (0.2)	26 (6.3)
Individual with diabetes (type not specified)	4 (0.8)	39 (9.4)
Caregiver/parent/guardian	0	38 (9.1)
Spouse/significant other	0	3 (0.7)
Friend	0	1 (0.2)
Nurse	2 (0.4)	9 (2.2)
Physician	3 (0.6)	6 (1.4)
Diabetes educator	0	11 (2.6)
Dietician	2 (0.4)	2 (0.5)
Researcher	2 (0.4)	4 (1.0)
Diabetes company	1 (0.2)	7 (1.7)
Diabetes company employee	0	22 (5.3)
Health care organization	12 (2.4)	6 (1.4)
Other/unknown	471 (94.2)	65 (15.6)

## Discussion

### Principal Findings

We describe the frequency, timing, and location of diabetes-related tweets on Twitter using a large comprehensive dataset of 10% of all tweets over a 2-year period. The large and increasing volume of tweets demonstrates that social media is a growing and robust medium where communications related to diabetes are taking place; in addition, the location of tweets indicates that they are happening at a global scale.

In terms of participants on Twitter, we did not identify clear diabetes stakeholders from our random sampling of users from the pool of all diabetes-related tweets. However, when we focused on users from the #dsma community, we did find a significant proportion of patients with type 1 diabetes represented, demonstrating that they are using the medium and hashtag to communicate with a larger virtual community about diabetes during their weekly tweetchat. We found that a very small percentage of participants were health care providers, which may be consistent with the fact that #dsma is a patient-focused chat but may also underscore the fact that physicians are reluctant participants or prefer to hide their physician or health care provider identities with regard to social media [9].

### Strengths and Limitations

Strengths of our study include the ability to extract 10% of all tweets from the Twitter database over an extended time period, the use of geolocated data, and the examination of the identity of participants who are tweeting. However, we must also acknowledge limitations of our study. Because we only had access to a 10% sample, we could not perform social network analysis of the diabetes community on Twitter. We also recognize that there may be limitations with using hashtags to define a community.

### Conclusions

Twitter is increasingly becoming a space for online conversations about diabetes. Further qualitative and quantitative content analysis is needed to understand the nature and purpose of these conversations.

## Abbreviations

API: application programming interface
DSMA: Diabetes Social Media Advocacy
